# Virus–Host Cell Interactions

**DOI:** 10.3390/cells11050804

**Published:** 2022-02-25

**Authors:** Thomas Hoenen, Allison Groseth

**Affiliations:** 1 Laboratory for Integrative Cell and Infection Biology, Institute of Molecular Virology and Cell Biology, Friedrich-Loeffler-Institut, 17493 Greifswald, Germany; 2 Laboratory for Arenavirus Biology, Institute of Molecular Virology and Cell Biology, Friedrich-Loeffler-Institut, 17493 Greifswald, Germany

As obligate intracellular parasites, viruses are intimately interconnected with their host cells. Virus–host cell interactions allow viruses to exploit cells for their own purposes, but they also provide a means for the host cell to combat virus infection. This close connection between host and viral processes means that the scientific fields of cell biology and virology have often inspired each other.

In particular, many discoveries in cell biology have been made possible by the study of viruses, while, at the same time, our fundamental understanding of the virus life cycle is inherently rooted in the principles of cell biology. By examining cellular responses to infection, we can gain insights regarding the mechanisms associated with the restriction of virus infection or, in cases where control is ineffective, pathogenesis. Such knowledge is a prerequisite for the successful modulation of these responses to develop host-directed therapies for the control of viral infections. Furthermore, from a practical point of view, virus–host cell interactions also provide important targets for the development of indirectly acting antivirals, which have a reduced likelihood to develop resistance due to their reliance on host cell components.

This Special Issue of *Cells* compiles both review papers discussing the current state of our knowledge regarding important emerging themes related to virus–host cell interactions, and research articles reporting new discoveries in this scientific area. These publications cover various aspects of the virus life cycle, from virus entry, uncoating, and virus replication in specialized replication compartments, all the way to virus particle production. They also touch on the complex interplay of viruses with the immune system, and current topics such as the modification of viral RNAs.

One of the first interactions of a virus with its host cell is during the entry process, which is generally facilitated by the interaction of a virus surface protein with a cellular receptor, and sometimes by additional interactions with cellular attachment factors, in order to facilitate virus uptake. Here, a mechanism that is increasingly recognized as being exploited by diverse virus families is apoptotic mimicry, which in its canonical form involves the exposure of phosphatidylserine in the outer leaflet of the viral membrane. This exposed phosphatidylserine can then be recognized by host cell proteins, such as those of the T-cell immunoglobulin and mucin domain (TIM) family. Kirui et al. [1] demonstrate that this mechanism is also used by Chikungunya virus. Their study also acts as an important reminder regarding the importance of working with authentic viruses, since their results with authentic Chikungunya virus show marked differences to previous results that were obtained based on experiments with pseudotyped particles alone.

In a second study on virus entry, but from the perspective of the role that virus entry receptor expression can play in directing infection outcome in different tissues, DeBuysscher et al. [2] show that Nipah virus efficiently replicates in human smooth muscle cells, even though these cells lack the canonical Nipah virus receptor ephrin B2. Furthermore, this lack of ephrin B2 appears to protect these cells from cell–cell fusion and cytopathic effects seen in other target cells that express this host factor, and suggests that smooth muscle cells might play an important role in pathogenesis by harboring and amplifying viruses that then infect and damage neighboring endothelial cells.

After entry, the next hurdle that many viruses have to overcome in order to establish a successful infection is the uncoating of the virus genetic material to facilitate its release. In a featured review on Influenza virus uncoating, Moreira at al. [3] discuss how the virus utilizes host cell pathways for this purpose. In particular, they highlight the role of a number of cellular factors, such as ubiquitin, histone deacetylase 6 (HDAC6), and transportin 1, and discuss potential contributions of these proteins for the uncoating process of other RNA viruses, as well as their potential as targets for broad-spectrum-antivirals.

For viruses that replicate in the cytoplasm, genome replication and viral transcription are increasingly being appreciated to take place in specialized replication organelles. For positive-strand RNA viruses, these are predominantly membranous structures. In contrast, for a growing number of negative-strand RNA viruses, viral RNA synthesis has been shown to be localized in inclusion bodies that are not delineated from their surroundings by membranes. Two reviews highlight the progress that is being made in understanding both of these types of replication organelles. Nguyen-Dingh and Herker [4] focus on the membranous replication organelles induced by positive-strand RNA viruses, whereas Dolnik et al. [5] discuss exciting recent progress in our understanding of negative-sense RNA virus replication structures as liquid organelles, i.e., compartments that are held together by liquid–liquid phase separation, rather than by a surrounding membrane.

Within these replication organelles, transcription leads to the generation of viral mRNAs. However, what has remained underappreciated in virology is that, in many cases, these mRNAs can be modified by the addition of methyl groups by host cell proteins. In the second featured review of this Special Issue, Courtney [6] provides a comprehensive overview regarding the current state of our knowledge with respect to this emerging topic, highlighting not only recent scientific advancements in understanding the functional implications of RNA modifications, but also giving an overview of the available methods for exploring them in a viral context.

Finally, in order to complete their life cycle, viruses have to exit their host cell, and this process is often intimately interlinked with the subversion of cellular host factors. In the case of non-segmented negative-sense RNA viruses, particle production is frequently driven by a dedicated viral matrix protein. Focusing on the Ebola virus matrix protein VP40, Paparisto et al. show that the process of particle production is inhibited by the cellular protein HECT and RCC1-like containing domain 5 (HERC5) [7]. However, interestingly, the mechanism for this does not appear to be an inhibition on the level of matrix protein function, but rather involves the depletion of mRNAs encoding for VP40. Consequently, this study emphasizes not only the role virus host–cell interactions play in supporting the virus life cycle, but also the role that antiviral factors can play in inhibiting key viral processes.

Of course, host–pathogen interactions involved in antiviral control not only occur at the level of specific antiviral cellular factors or cellular antiviral responses, but can also include a broader range of interactions with the immune system. This is highlighted in a comprehensive review by Muralidharan and Reid, in which they illuminate the complex roles of neutrophils during arbovirus infections [8]. Using examples from a wide range of arboviruses, including Zika virus, Dengue virus, West Nile virus, and various alphaviruses, they highlight not only the beneficial roles that neutrophils can play, but also their sometimes-detrimental roles in augmenting disease pathology.

An equally complex but currently underappreciated topic is that of pathogen–host interactions in the context of coinfections involving several disease agents, and particularly co-infections of viruses and bacteria. In this context, a study by Nickol et al. [9] demonstrates the impact of coinfection with Influenza virus and methicillin-resistant *Staphylococcus aureus* on the expression of bacterial virulence factors as well as on the infected host, particularly regarding its cytokine response and the integrity of the alveolar-capillary barrier. This work provides mechanistic support for the clinical observation that severe influenza infections are frequently complicated by bacterial coinfections.

Overall, this Special Issue provides examples of the crucial role that virus–host cell interactions play in the biology of a diverse range of viruses, and highlights the importance of better understanding such interactions in order to be better able to combat virus infections and the mechanisms that contribute to pathogenesis and disease.
